# A pool of peptides extracted from wheat bud chromatin inhibits tumor cell growth by causing defective DNA synthesis

**DOI:** 10.1186/1747-1028-8-11

**Published:** 2013-08-06

**Authors:** Loretta Mancinelli, Teresa Secca, Paula M De Angelis, Francesco Mancini, Matteo Marchesini, Cristiano Marinelli, Lanfranco Barberini, Francesco Grignani

**Affiliations:** 1 Department of Cellular and Environmental Biology, University of Perugia via Pascoli, 06123, Perugia, Italy; 2 Clinic for Diagnostics and Intervention, Oslo University Hospital-Rikshospitalet, Oslo, Norway; 3 Department of Clinical and Experimental Medicine, University of Perugia, Faculty of Medicin, S.Andrea delle Fratte, 06132, Perugia, Italy

**Keywords:** DNA damage, Chromatin peptides, H2AX, G2 checkpoint, BrdUrd comet

## Abstract

**Background:**

We previously reported that a pool of low molecular weight peptides can be extracted by alkali treatment of DNA preparations obtained from prokaryotic and eukaryotic cells after intensive deproteinization. This class of peptides, isolated from wheat bud chromatin, induces growth inhibition, DNA damage, G2 checkpoint activation and apoptosis in HeLa cells. In this work we studied their mechanism of action by investigating their ability to interfere with DNA synthesis.

**Methods:**

BrdUrd comet assays were used to detect DNA replication defects during S phase. DNA synthesis, cell proliferation, cell cycle progression and DNA damage response pathway activation were assessed using 3H-thymidine incorporation, DNA flow cytometry and Western blotting, respectively.

**Results:**

BrdUrd labelling close to DNA strand discontinuities (comet tails) detects the number of active replicons. This number was significantly higher in treated cells (compared to controls) from entry until mid S phase, but markedly lower in late S phase, indicating the occurrence of defective DNA synthesis. In mid S phase the treated cells showed less 3H-thymidine incorporation with respect to the controls, which supports an early arrest of DNA synthesis. DNA damage response activation was also shown in both p53-defective HeLa cells and p53-proficient U2OS cells by the detection of the phosphorylated form of H2AX after peptide treatment. These events were accompanied in both cell lines by an increase in p21 levels and, in U2OS cells, of phospho-p53 (Ser15) levels. At 24 h of recovery after peptide treatment the cell cycle phase distribution was similar to that seen in controls and CDK1 kinase accumulation was not detected.

**Conclusion:**

The data reported here show that the antiproliferative effect exhibited by these chromatin peptides results from their ability to induce genomic stress during DNA synthesis. This effect seems to be S-phase specific since surviving cells are able to progress through their normal cell cycle when the peptide fraction is removed from the culture medium. It is likely that the subsequent apoptosis is a consequence of the failed attempt of the tumour cells to repair the DNA damage induced by the peptides.

## Background

Alkali treatment of deproteinized DNA from mammalian and plant cells (calf thymus, bull spermatozoa, trout testis, wheat germ, pea and wheat bud) dissociates a pool of peptides of about 1000 Da with strongly related amino-acid composition. Their affinity for DNA is pH-dependent and is lower at alkaline pHs [1,2]. We previously reported that this class of peptides is able to inhibit RNA transcription in cells and *in vitro* reconstituted systems and to decrease cell growth of several tumour cell lines [3,4]. The removal of this fraction by alkaline buffer from the DNA of normal cells increases the DNA template capacity, but this effect is practically absent for the DNA of several cancer cell lines [5]. Accordingly, their concentration in the chromatin of cancer cells is lower than that present in the chromatin of the corresponding normal cells [6] so it is likely that they exert a role in controlling the mechanism of cell transformation. Studies aimed at investigating their effects on cell proliferation showed that this pool of peptides induced accumulation of cells in G2 phase, DNA damage and apoptosis in HeLa cells. We also reported that they activate the G2 checkpoint pathway, the regulatory mechanism that prevents entry of the cells into mitosis in response to defective DNA replication. The growth rate inhibition is obtained when the cells are treated during S phase only [7]. We therefore hypothesize that the antiproliferative effect exhibited by these chromatin peptides results from their action during DNA synthesis. In this study we wanted to investigate the effect of these peptides on the progression of DNA synthesis and evaluate the cellular response to the induced DNA damage.

## Results

**Figure 1:**
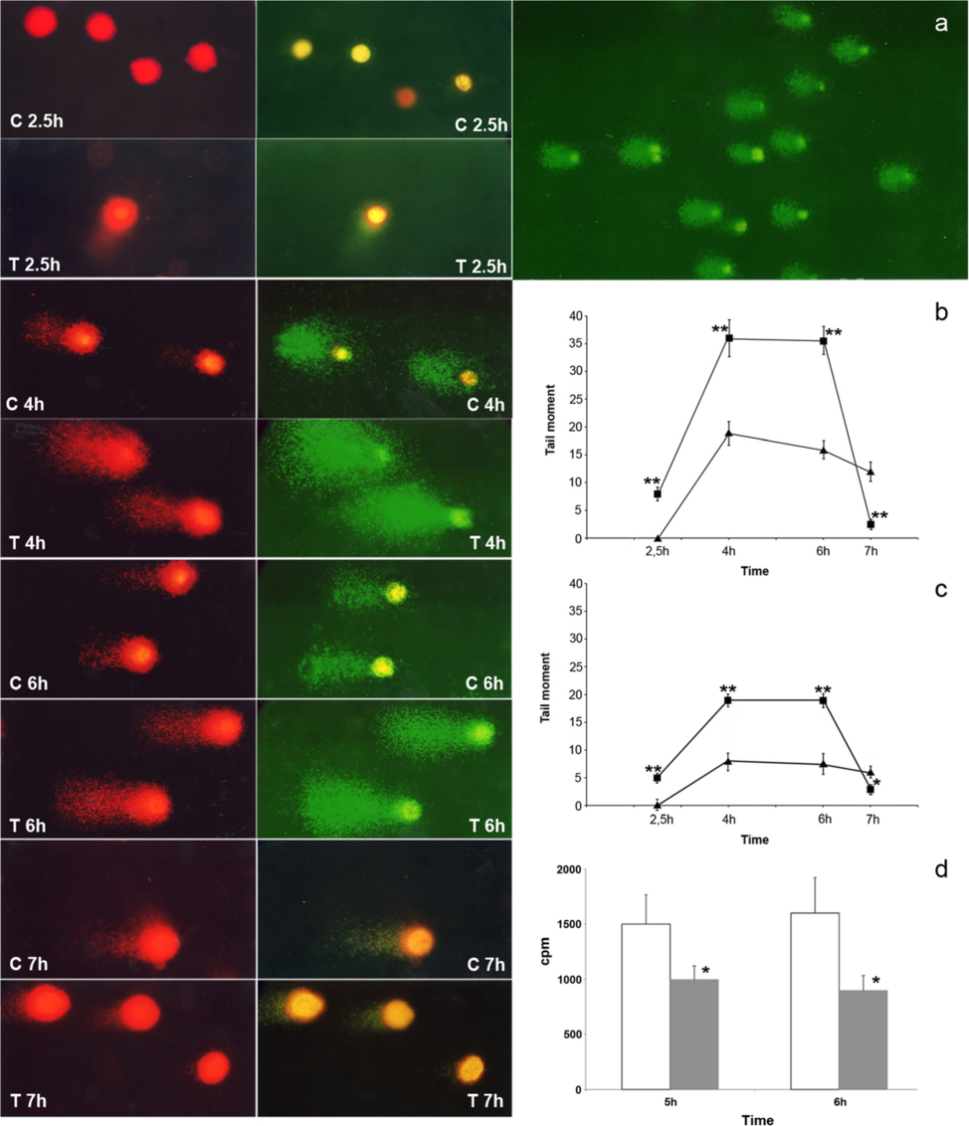
**Analysis of DNA synthesis in S phase synchronized HeLa cells.** Left panel: images of the BrdUrd Comet assay performed at 2.5, 4, 6 and 7 hours from the beginning of DNA synthesis. The pictures, obtained by fluorescence microscopy show identical comet fields with FITC (green) and propidium iodide staining (red). C: control cells. T: treated cells. Right panel: Quantification of DNA synthesis at different time points from the beginning of S phase. **(a)**: representative image of BrdUrd-Comet assay (INGR ×150, FITC staining), the percentage of tail DNA shared uniform distribution among the various comet formations. Tail moment measured following FITC **(b)** and PI **(c)** stainings, respectively. Controls (triangles); treated (squares). Results shown are the mean ± SE of 25 cells. **(d)**: Thymidine incorporation per cell at 5 and 6 hours from the beginning of S phase. Control cells (black), treated cells (grey). The error bars show the standard deviation obtained from three independent experiments. Student’s test. *p < 0.05 **p < 0.01.

Cell cycle arrest and apoptosis were induced in HeLa cells after incubation with a pool of peptides extracted from wheat bud chromatin [7]. A mechanism of action was proposed in which the inhibition of cell growth results from their ability to affect DNA replication. In order to provide a more detailed picture of their action on this process, we performed the BrdUrd Comet assay in synchronized HeLa cell cultures during the DNA synthesis. S phase cells were obtained by the double thymidine block that arrests the cells at the G1/S boundary. The removal of thymidine by replacement with normal medium induces the onset of S phase. DNA synthesis was analyzed by determination of ^3^H thymidine incorporation into DNA at 1 hour intervals. The time course of incorporation indicates that ^3^H thymidine uptake initiates shortly after the removal of the thymidine block, reaches the maximum after 6 hours and drops at 8 hours. This pattern demonstrates the occurrence of synchronization since cells not subjected to the thymidine block show a constant increasing rate of ^3^H thymidine incorporation (data not shown). Immediately after the removal of the thymidine block, the cells were incubated with the peptide pool for 2.5, 4, 6 and 7 hours, while replicating DNA was labelled by adding the thymidine analogue BrdUrd to the culture medium. The cells were then collected and subjected to the comet assay. The label incorporation was detected, following electrophoresis of the cells, by immunological assay. Figure 1 shows the localization of such label at different time points during DNA synthesis and the quantification of the DNA fragmentation (PI staining) and BrdUrd labelling (FITC staining) within the comet tails. In the control cells, at the beginning of the DNA synthesis (2.5 hours from the removal of thymidine block), little DNA fragmentation was detected. After 4 hours from the onset of the DNA synthesis, DNA fragmentation was detected and the BrdUrd label was localized close to the strand discontinuities, constituting the comet tails. In late DNA synthesis, 6 and 7 hours from the onset of the DNA replication, the level of the DNA strand breaks and BrdUrd incorporation in the tails decreased. In the treated cells, from the beginning (2.5 time point) till mid S phase, the levels of DNA fragmentation and tail labelling were significantly higher than in the controls. They reached the maximum value after 4 hours from the onset of the S phase, remained roughly the same at 6 hours and sharply decreased at the 7-hour time point. We determined the DNA synthesis activity at 5 and 6 hours from the beginning of the S phase by pulse labelling the cells with ^3^H thymidine for 30 min. The radioactivity incorporation per cell was lower in the treated cells respective to the control cells (Figure 1d). These data support our hypothesis of an action of this pool of peptides in the DNA replication process.

**Figure 2:**
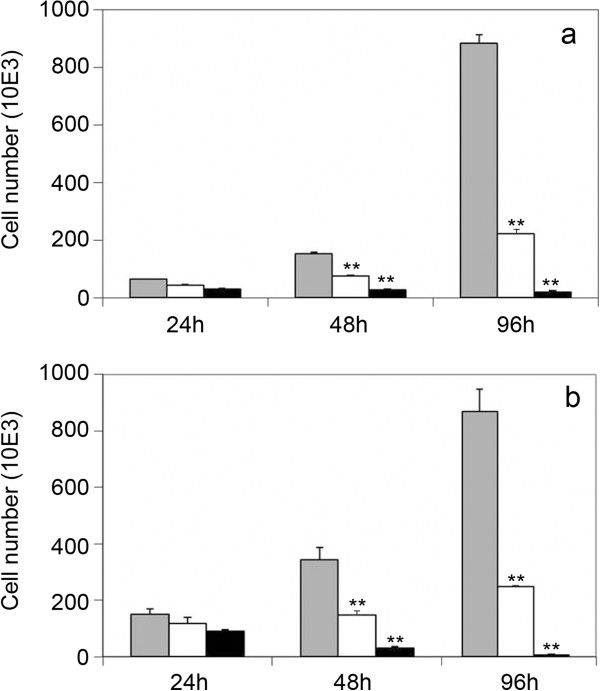
**Cell growth inhibition of tumour cell lines induced by the peptide fraction.** HeLa **(a)** and U2OS **(b)** cells were treated with 1 μg/ml (white) and 2 μg/ml (black) of peptide pool for 1, 2 and 4 days. Gray bars represent untreated controls. Error bars show the standard deviation obtained from three independent experiments. Student’s test. *p < 0.05 **p < 0.01.

**Figure 3:**
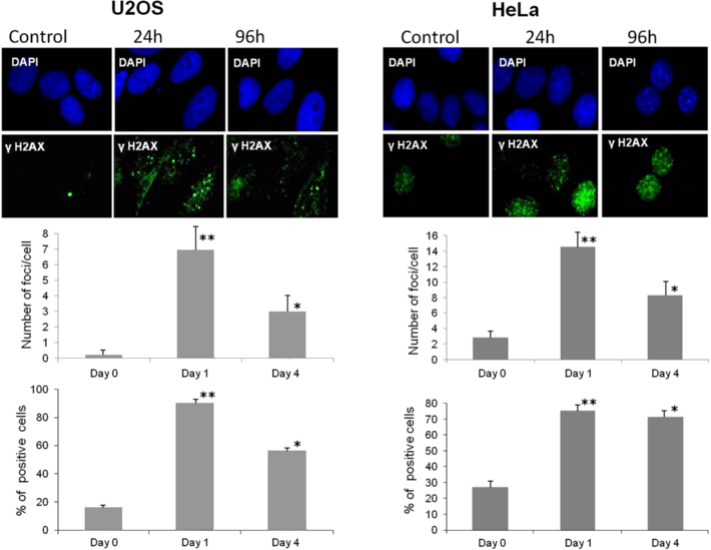
**Immunofluorescence of U2OS and HeLa cells treated with the peptide pool (2 μg/ml) for 24 and 96 hours.** Top panel: confocal microscope images of phospho-foci stained with γH2AX antibody (green) and DAPI (blue). Lower panel: percentage of γH2AX positive cells, and average number of γH2AX foci per cell as scored from 100 cells. Error bars show the standard deviation obtained from four independent experiments. Student’s test. *p < 0.05 **p < 0.01.

**Figure 4:**
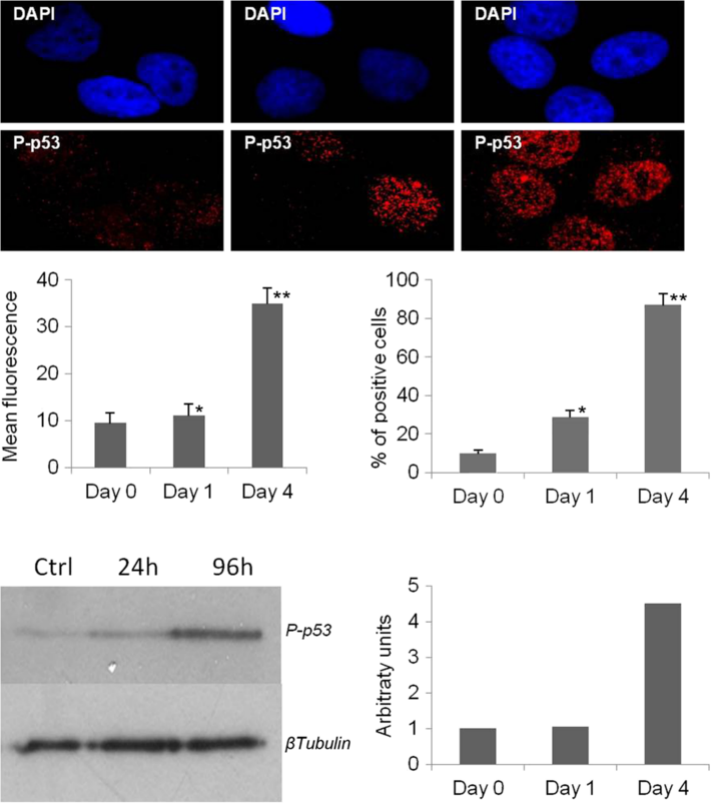
**Analysis of p53 expression in U2OS cells treated with the peptide pool for 24 and 96 hours.** Immunofluorescence of U2OS cells stained with DAPI (blue) and p53 antibody (red). The percentage of γH2AX positive cells, derived from five different fields, and the average number of γH2AX foci per cell, as scored from 100 cells, are reported. Lower panel: Western blot analysis image and densitometric quantization of phospho-p53 level following normalization with tubulin.

In response to DNA replication stress, checkpoint proteins are activated to trigger the DNA repair machinery. A critical component of the signaling pathway is represented by the histone H2AX that, in its phosphorylated form γH2AX, specifically attracts proteins leading to the formation of nuclear DNA repair foci [8]. In order to investigate the induction of the DNA repair pathway following replication stress, we checked the expression of γH2AX in peptide treated cells. We tested two cell lines, HeLa and U2OS, that differ in their expression of the p53 protein. While in HeLa p53 is inactivated by the E6 protein encoded by the HPV genes [9], in U2OS it can be induced in the mediation of the cellular response to genotoxic stress. We first checked whether the peptide pool exerted antiproliferative effects also in U2OS cells with functional p53. We obtained (Figure 2) a level of growth inhibition in treated U2OS cultures comparable to that obtained for HeLa cells. In Figure 3 we show that the peptide treatment increases the percentage of γH2AX positive cells and the average number of γH2AX foci per cell in both cell lines. The involvement of p53 has been also investigated in U2OS cells by measuring the expression of phospho p53 (Ser-15) which is induced during the cellular response to DNA damage. The exposition of U2OS cells for 4 days to the peptide pool results in an increase of 4.5 times the control level of the expression of active p53. In agreement with Western Blot analysis, immunofluorescence results revealed a higher percentage of phospho-p53 (Ser-15) positive cells in the treated culture with a higher average level of fluorescence intensity per cell with respect to the control cells (Figure 4). Several small molecules with the ability to reactivate mutant p53 have been reported [10]. We therefore checked the possibility that in HeLa cells the treatment could interfere with the p53 degradation to recover its function, but no expression of phospho p53 was detected in this cell line (data not shown).

**Figure 5:**
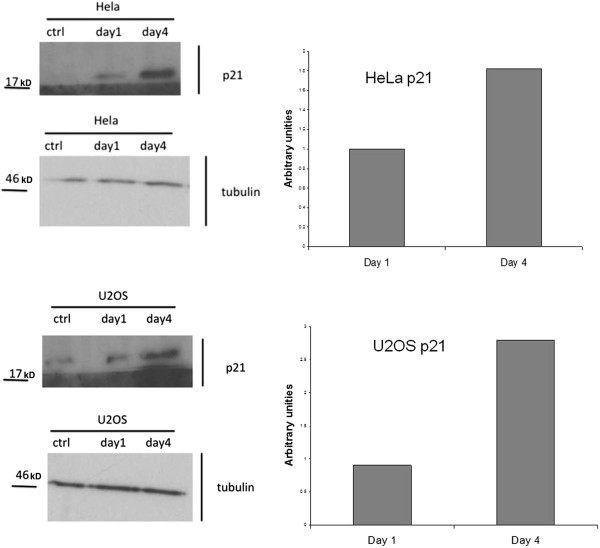
**Analysis of p21 expression in Hela and U2OS cell lines.** Western Blot analysis was performed in the cells treated for 24 and 96 hours with the peptide fraction. Densitometric quantification of p21 levels were obtained following normalization with tubulin. The molecular weight standards are shown on the left.

We also investigated the expression level of the p21 protein, an inhibitor of CDK-cyclin complexes, since it has been reported to arrest cell cycle progression in response to DNA damage. Western Blot analysis showed that the level of p21 expression in treated Hela and U2OS cells is higher compared to the controls (Figure 5). This suggests the involvement of p21 protein in the pathway adopted by the cells to induce G2 phase block that occurs after a defective DNA replication in the cells treated with the peptide pool.

**Figure 6:**
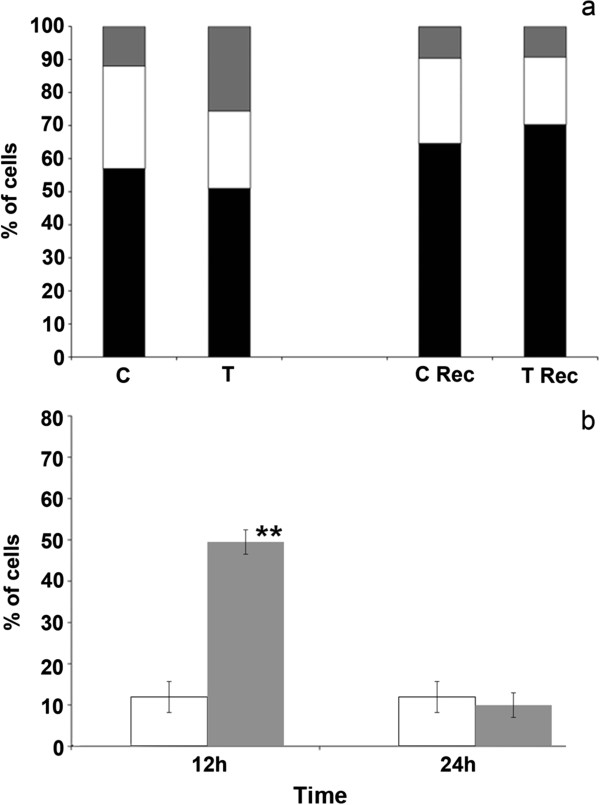
**Flow cytometry analysis of the cells treated with the peptide pool. a**: Percentage of cells in G1 (black), S (white) and G2 (grey) phases after 12 hours of peptide treatment (T) and after 24 hours of recovery (36-hour time point) in fresh medium (T rec.). C and C rec. represent the corresponding controls. The experiment shown is representative of three independent experiments. **b**: percentage of cells expressing CDK1cyclinB complex following 12 hours of treatment and 24 hours of recovery (36-hour time point) in normal medium. Control cell (black), treated cells (grey). Error bars show the standard deviation obtained from three independent experiments. Student’s test. *p < 0.05 **p < 0.01.

We have previously reported that cell growth inhibition is obtained when synchronized cells are treated during S phase only [7]. In order to assess the selectivity of the peptide action on the replication process, we evaluated the ability of the surviving cells to undergo a normal cell cycle progression after the removal of the peptide fraction from the culture medium. Unsynchronized HeLa cells were exposed to the peptide pool for 12 hours, then the pool was removed from the medium and cells were grown in normal medium for 24 hours. Cell cycle analysis and CDK1 expression level were measured at each time point. 12 hours of treatment induced G2 arrest and an accumulation of CDK1 kinase in the G2 cells. After 24 hours of recovery the cell distribution in the different phases of the cell cycle was the same as in the untreated cells. As shown in Figure 6, G2 arrest and CDK1 kinase accumulation were not detected. Taken together, these data demonstrate that the peptide pool exerts a selective action on S phase cells inducing a subsequent G2 arrest and apoptosis. The remaining cell population, unaffected, can continue cell cycle progression.

## Discussion

A class of DNA binding peptides is reported to be able to control HeLa cell proliferation by causing arrest of cell cycle progression in G2 phase and apoptosis. The inhibition of cell growth is obtained when synchronized cells are treated during S phase only and is accompanied by DNA damage, increase in the expression of the active Chk1 kinase and accumulation of the inactive CDK1 kinase in G2 cells. These findings indicate that the success of this pool of peptides in arresting the tumour cell cycle could result from their ability to affect DNA replication and as a consequence, to activate the checkpoint pathway that prevents mitosis of cells with damaged DNA. In order to provide more evidence supporting this mechanism, we wanted to assess the defective DNA replicative integrity and the possible induction of DNA repair processes in cells exposed to this class of peptides in the present study.

We performed the BrdUrd-Comet assay on S phase-synchronized HeLa cells at various time points from the onset of DNA synthesis. In the control cells, the DNA replicating label is initially detected within the tails of the comets, close to the DNA fragments that are generated at the leading and lagging strand growth points. Its amount within the strand discontinuities provides evidence of the number of the initiation sites of DNA synthesis [11]. They increased until mid S phase and decreased in the second half of DNA synthesis. After treatment of the cells with the peptide pool, the numbers of active sites present until mid S phase were higher respective to the control cells, while in late S phase the numbers sharply decreased and were lower than those reported for control cells.

It is now accepted that in eukaryotic cells, replication origins are activated at different times through S phase following a stochastic program. The time dependent rate of initiation (number of initiation per time unit per unit of length of unreplicated DNA), i(t), increases until mid S phase and sharply decreases before the end. The increasing number of active replication sites during a normal DNA synthesis, as S phase progresses, is due to an increased efficiency of origin firing, while their decrease is a consequence of the mechanism of self limiting action of the fork density [12]. The behaviour of control cells in our BrdUrd Comet assay is therefore consistent with this temporal regulation of origin activation. In treated HeLa cells the number of active replicons, in early and mid S phase, is higher than in the controls while it is lower in late S phase. It has been reported [11] that the BrdUrd Comet method detects strand discontinuities in recently replicated domains of DNA caused during replication and that the arrest of replicative polymerase at DNA lesions prolongs the duration of these breaks. Therefore it is likely that the peptide treatment induces stalled replication forks and a delay in the maturation process of DNA, which are responsible for the reported higher amount of BrdUrd labelling in the comet tails. Accordingly, despite the higher number of active replicons in the treated cells respective to the controls, the amount of ^3^H thymidine incorporation per cell in mid S phase, was lower in agreement with an arrest in DNA synthesis. It is likely that such arrest in DNA maturation is responsible for the sharp decrease in the number of active replicons reported in treated cells in late S phase, in agreement with the self limiting mechanism mentioned above.

Alternatively, the observed differences in BrdUrd labelling in the comet tail between treated and control cells could be due to the action of the peptide pool in increasing the efficiency of origin firing. It has been reported [13] that a higher number of active sites of replication could be explained by a better availability of some limiting factor for DNA replication. The drop in the number of active replicons detected in treated cells at the end of the S phase, according to the stochastic model of replication origin activation, is due to the negative correlation between fork density and origin firing. Although further experiments are needed to assess the mechanism of action of the peptide pool, the data reported in this work demonstrate that the peptide acts during early S phase by inducing defective replication, DNA damage and early arrest of DNA synthesis. Consistent with these findings, we previously obtained G2 checkpoint pathway activation and a shortening of S phase length after peptide treatment in HeLa cells.

p53 protein plays a central role in the cellular response to DNA damage [14], therefore we investigated whether the peptide pool failed to inhibit cell growth in p53 positive tumour cells. We obtained an antiproliferative effect in U2OS cells comparable to the one obtained in HeLa cells where p53 is inactivated, thus showing the possibility for this class of peptides to affect the proliferation of tumor cell lines with differing p53 functionalities. We also found expression of γH2AX in both cell lines treated with the peptide pool, which demonstrates the induction of the cellular response to the DNA damage. In U2OS cells this process involves activation of p53, which is reflected by the phosphorylation of Ser15, the consensus site for a series of kinases, such as ATM/ATR/DNA-PK, activated during the DNA repair pathway [15]. We also obtained an increase in the expression of p21 in HeLa and U2OS treated cells, which demonstrates the involvement of this protein in the cellular activated response, by its action in arresting cell cycle progression to allow cells to repair DNA damage [16]. While in U2OS cells p21 induction is a consequence of p53 activation, in HeLa cells, increase in expression of p21 is due to a p53-independent mechanism.

Using the same procedure, we have extracted this class of peptides from the chromatin of different types of mammalian and plant cells. Their ubiquitous presence is consistent with their involvement in a conserved mechanism such as the control of the DNA synthesis process. We also showed that this effect is highly selective, since the cells that survived the treatment are able to recover their normal cell cycle progression in the absence of the peptide pool. Therapeutic selectivity and low toxicity are the major goals in designing novel anticancer drugs. In this context, peptide based therapies offer some important advantages over other chemotherapy molecules such as high affinity and specificity for target molecules, low toxicity and good bio-availability [17]. Experiments are in progress to identify the pool sequence(s) responsible for the reported effects.

Mass spectrometry analysis has allowed identification of some structures with sequences very similar to those present in the phosphorylation sites for CKII kinase of many transcription factors such as RNA pol.II Sub.215 KD, PML, HPV18E7, Human Myc, Chicken Myb, Human Rfx, human Ubf1, RNA Pol.Sigma 70 factor [18]. However, unpublished preliminary evidence suggests that the reported effects on cell growth result from the action of various sequences or from a complex of different molecules. Further studies are needed to improve HPLC purification and mass spectrometry analysis in order to identify new molecules belonging to the pool.

## Conclusions

We have studied the mechanism of action of a highly-conserved class of DNA binding peptides with the ability to inhibit tumor cell growth. We report here that the main action of these antiproliferative peptides is impairment of the replicative integrity causing an early arrest of DNA synthesis. The resulting genomic stress induces the activation of the checkpoint pathway to allow the cells to repair the DNA damage. It is likely that the subsequent apoptotic process is a consequence of the inability of the tumour cells to repair the DNA damage and re-enter the cell cycle. It is possible to speculate that tumour cells, characterized by a high replicative activity and a defective DNA repair machinary, are the main target for the antiproliferative effect of our chromatin peptides. Studies directed to this hypothesis may be helpful in providing a potential therapeutic approach for the treatment of cancer.

## Methods

### Purification of peptides

The purification of peptides from wheat bud chromatin has been performed following the procedure previously described [2]. Briefly, the chromatin was purified at slightly acidic pH and then subjected to alkaline extraction. The macromolecules were removed by centrifugation after the treatment with two volumes of methanol, overnight at 4°C. The supernatant was recovered and the peptides were purified by gel filtration chromatography at Ve/V0 = 2.

### Cell culture

Human osteosarcoma U2OS and cervical cancer HeLa cell lines were obtained from the American Type Culture Collection (ATCC; http://www.atcc.org) and tested for mycoplasm by using Mycoplasma Detection Kit (Roche Germany). They were grown in 25 cm^2^ flasks in 5 ml of Dulbecco’s Modified Eagle Medium with 10% fetal calf serum, penicillin at 100 units/ml, streptomycin at 100 μg/ml and 2 mM glutamine (Invitrogen Paisley UK) at 37°C humidified atmosphere of 5% CO_2_/95% air. The peptides were added to the culture medium 24 hours after cell seeding (25,000 cells/ cm^2^). Cell numbers were evaluated by direct counting using a hemocytometer, and cell viability was assessed by trypan blue exclusion. Cell synchronization was obtained by a double thymidine block as described by Kozaki et al. [19]. 6 h following seeding, the cells were exposed to 2 mM thymidine (Sigma) for 16 h, to normal medium for 8 h and to 2 mM thymidine for 16 h. The subsequent replacement with normal medium released the cells at the G1/S boundary.

### Cell cycle analysis

The cells were trypsinized and analyzed, as previously described [7], for DNA content using a FACSCalibur laser flow cytometer (Becton Dickinson Immunocytometry Systems, San Jose, CA). Pulse-processed fluorescence signals were used to exclude doublets and aggregates from analyses. Ten thousand events were acquired for each sample. Percentages of cells in the G_1_, S, and G_2_ M phases of the cell cycle were quantified using WinCycle software (Phoenix Flow Systems, San Diego, CA).

### Cdc2 immunofluorescence analysis

Cells (2 × 10^6^) were trypsinized, washed twice with 10 mM phosphate buffer (PBS) and fixed in 90% ethanol. After washing in PBS the pellet was treated with PBS plus 0.1% Triton X and resuspended in 100 μl PBS containing 0.1% Triton X100 plus 3% non-fat dry milk. The primary antibody (cdc2, Santa Cruz Biotechnology) was added to a final concentration of 2 μg/ml, and allowed to incubate on ice for 1 hour. Suspensions were then washed in PBS containing 0.1% Triton X100. Pellets were resuspended and incubated with PBS containing 0.1% Triton X100, 3% non-fat dry milk, and biotinylated horse-anti-mouse IgG (1:50 dilution of stock, Vector Labs, Burlingame, CA) for 30 minutes. Suspensions were then washed in PBS containing 0.1% Triton X100. Pellets were resuspended and incubated with PBS containing 0.1% Triton X100, 3% non-fat dry milk, and streptavidin-FITC (1:50 dilution of stock, Amersham Biosciences, UK) for 30 minutes. Suspensions were again washed in PBS containing 0.1% Triton X100, and then resuspended in 5 μg/ml propidium iodide made up in PBS containing 0.1% Triton X100, to stain DNA. Samples were then run on a FACSCalibur flow cytometer, and correlated analyses of cell cycle proteins and DNA content (to allow for cell cycle analysis) were generated.

### Incorporation of 3H thymidine into DNA

Synchronized cells were grown at a cell density of 10000/cm^2^. At different time points from the removal of the thymidine block, the cells were incubated with 1 μCi/ml of ^3^H-thymidine (Amersham, Buckinghamshire, UK) for 30 min. Radioactivity incorporation was measured by liquid scintillation counting as previously described [7].

### BrdUrd-comet assay

The cells were seeded at a 10000/cm^2^ cell density and synchronized by the double thymidine block. Immediately after their release in S phase, the cells were incubated with the peptide pool and the thymidine analogue BrdUrd (100 μM/ml) for 2.5, 4, 6 and 7 hours. The cells were then harvested by trypsinization, washed twice with PBS and subjected to the alkaline comet assay following the procedure already described [7]. After electrophoresis, the slides were rinsed in neutral buffer (0.4 M Tris-HCl pH 7.5) and the incorporation of BrdUrd was immunologically detected according to the procedure reported by Mc Glyn et al. [11]. Briefly, 25 μl of mouse monoclonal anti-BrdUrd (10 μg/ml, Sigma) was added to each slide and allowed to incubate in the dark, at room temperature, for 1 h.

The primary antibody was removed by three washes with PBS and one wash with PBS plus 0.1% BSA. Each slide was then incubated with 25 μl of secondary anti-body (5 μg/ml sheep anti-mouse IgG, Sigma) for 1 h in the dark at room temperature and rinsed as before. The DNA was counterstained with propidium iodide (0.75 μl/ml, Sigma) and the comet formations were analyzed using the fluorescence microscope LEICA DMRB. Tail moment was calculated by analysing 25 cells for each time point.

### DNA damage analysis by immunofluorescence staining

Cells (0.03*10^6) were plated on a coverslip in a 12-well plate, and the peptidepool was added to the culture medium 24 hours after cell seeding. When indicated at different time points, cells were washed with Dulbecco’s Phosphate Buffered Saline (DPBS, Lonza Walkersville, Inc. USA #17-512 F) 3x and fixed with 4% paraformaldehyde (Sigma-Aldrich #P6148-500G) in PBS. Cells were permeabilized using 0.5% Triton X-100 in PBS for 5 min at RT. The blocking solution used was 5% Bovine Serum Albumin in PBS for 30 min at RT. The slides were incubated with phospho (γ)-H2AX (Ser 139 Millipore) and phospho-p53 (Ser15 Cell Signaling) primary antibodies diluted in 5% BSA in PBS for 1 hour at RT and then with anti-mouse Cy3 or Alexa 488 (Jackson Laboratory) secondary antibody in PBS 1% BSA for 45 min. Nuclei were stained with 4,6-diamidino-2-phenylindole (DAPI Sigma-Aldrich). Stained slides were mounted with Mowiol and analyzed using an Olympus microscope equipped with a CCD color camera (Hamamatsu). Imaging statistics were performed using ImageJ software (http://rsbweb.nih.gov/ij/). 100 cells/time point were evaluated for positive/negative staining, the number of foci/nucleus and signal intensity for both γH2AX and phospho-p53 proteins using different ImageJ plugins.

### Western blotting

Cells were lysed in Laemmli buffer and samples were electrophoresed on SDS-polyacrylamide gels. Separated proteins were transferred onto a nitrocellulose membrane, which was then incubated with the following antibodies: p21 (Santa Cruz), phospho-p53 (Ser15) (Cell Signaling) and β-tubulin (Sigma-Aldrich), each made up in 5% fat-free milk. Secondary antibodies were peroxidase-conjugated anti-mouse or anti-rabbit antibodies. Hybridizations were detected by enhanced chemiluminescence (ECL GE Healthcare, Amersham Bioscience, Piscataway, NJ, USA). WB signal quantification was assessed using ImageJ software.

## Competing interests

The authors declare that they have no competing interests.

## Authors’ contribution

LM designed the study and drafted the manuscript. TS made substantial contributions to analysis and interpretation of data and to the performance of the BrdUrd-Comet assay. PMD designed the flow cytometry experiments, performed data analysis and revision of the manuscript. FM, MM, CM performed DNA damage signaling experiments and contributed to data analysis. LB performed image analysis and construction of figures. FG directed DNA damage signaling experiments and revised the manuscript. All authors read and approved the final manuscript.
